# Thermostable and recyclable *Candida antarctica* lipase B immobilized on *Bacillus subtilis* using sortase technology

**DOI:** 10.1128/spectrum.01121-25

**Published:** 2025-10-27

**Authors:** Samira Ghaedmohammadi, Saghi Nooraei, Faezeh Ahrari, Faezeh Moosavi, Mehdi Mohammadi, Gholamreza Ahmadian

**Affiliations:** 1 Department of Biology, College of Sciences, Shiraz University 37551, Shiraz, Iran; 2 Department of System Biotechnology, National Institute of Genetic Engineering and Biotechnology (NIGEB) 48482, Tehran, Iran; 3 Bioprocess Engineering Department, Institute of Industrial and Environmental Biotechnology, National Institute of Genetic Engineering and Biotechnology (NIGEB) 48482, Tehran, Iran; Institute of Microbiology, Chinese Academy of Sciences, Beijing, China

**Keywords:** sortase-mediated surface display, CALB, *Bacillus subtilis*, enzyme immobilization, whole-cell biocatalyst

## Abstract

Bacterial surface display is a transformative strategy for enzyme immobilization, enhancing enzyme stability and reusability for industrial applications. This study presents a novel whole-cell biocatalyst by immobilizing *Candida antarctica* lipase B (CALB) on *Bacillus subtilis* cells via a sortase-mediated system. A genetically engineered CALB variant, incorporating a C-terminal cell wall-binding motif, was co-expressed with the sortase enzyme in *B. subtilis* WASD (ΔwprA ΔsigD) cells, facilitating stable and site-specific anchoring of CALB directly on the bacterial surface without the need for purification or chemical modification. The immobilized CALB exhibited superior performance compared to its free counterpart, displaying optimal activity at 37°C and pH 8.0, with significantly enhanced thermostability. It retained ~50% activity after nine reuse cycles and maintained >50% of its initial activity after 8 weeks of storage. Additionally, surface-displayed CALB efficiently hydrolyzed fish oil, selectively releasing omega-3 polyunsaturated fatty acids (EPA and DHA), demonstrating its potential for sustainable bioprocessing in the food, pharmaceutical, and nutraceutical industries. This work establishes a scalable and environmentally friendly approach for enzyme immobilization. By leveraging *B. subtilis* as a host, sortase-mediated anchoring provides a robust platform for developing thermostable, reusable, and industrially viable whole-cell biocatalysts.

**IMPORTANCE:**

This work presents a groundbreaking advancement in biocatalysis, addressing critical challenges in enzyme stability, reusability, and sustainability for industrial applications. This work directly addresses the industrial bottlenecks of enzyme instability, high turnover costs, and environmental impact. The sortase-mediated system offers a universal platform for displaying diverse enzymes, enabling tailored biocatalysts for sectors ranging from bioremediation to synthetic biology.

## INTRODUCTION

The expression of recombinant proteins fused with signal peptides to target specific locations on the cell surface, referred to as surface display, represents a transformative tool in biotechnology. By anchoring proteins to microbial cell surfaces, bacterial surface display serves as an efficient and cost-effective strategy for stabilizing enzymes and proteins, bypassing the need for extensive purification processes (1, 2). This dual approach of enzyme expression and immobilization not only streamlines production but also enhances enzyme stability, thermal resistance, and reusability—key attributes for industrial applications (3). Surface display technology is particularly valuable for its ability to present intracellular peptides and proteins on the exterior of living cells. These surface-displayed biomolecules often retain full biological functionality, making them highly versatile for applications such as live vaccine delivery, protein library screening, cell-based biodegradation, biosensors, and biocatalysis (4). Among these, the development of whole-cell biocatalysts, which integrate the catalytic activity of immobilized enzymes with the robustness of living cells, has gained significant attention. Such biocatalysts combine enzyme activity with cellular resilience, creating systems that are not only reusable but also easy to handle in industrial processes.

Lipase (EC 3.1.1.3, triacylglycerol acylhydrolase) is a quintessential enzyme in biotechnological applications. It catalyzes the hydrolysis of ester bonds in triacylglycerols and has proven indispensable in industries ranging from food processing and pharmaceuticals to biodiesel production and biopolymer synthesis (5). Lipases, particularly *Candida antarctica* lipase B (CALB), are highly valued for their broad substrate specificity, remarkable efficiency, and selectivity. Among the two isoforms of lipase produced by *Candida antarctica*—CALA and CALB—the latter is especially preferred for hydrolytic and transesterification reactions due to its superior catalytic properties (5, 6). However, like most free enzymes, CALB faces several limitations that hinder its industrial utility. These challenges include low stability under harsh reaction conditions, difficulty in recovery and recycling, and the high costs associated with its single-use applications. Immobilization has emerged as a powerful strategy to overcome these barriers. By attaching enzymes to various support materials, immobilization not only enhances their thermal and operational stability but also enables their reuse, reducing costs and environmental impact (7). For instance, Novozym 435, a commercially available immobilized CALB on an acrylic resin, exhibits exceptional thermal stability, retaining activity even at 100°C (8). Immobilization can create a microenvironment that stabilizes the enzyme’s active site, improving its resistance to thermal fluctuations. For instance, studies have shown that immobilized enzymes often retain higher activity and stability compared to their soluble counterparts when subjected to thermal stress (9). While immobilization on non-living surfaces such as silicon polymers (10), silver nanoparticles (11), and magnetic nanoparticles (12) has shown promise, these methods are often complex, expensive, and environmentally taxing. In contrast, immobilization on living microbial surfaces, such as those of *Saccharomyces cerevisiae* (13), *Pichia pastoris* (14, 15), *Bacillus* subtilis spores (16), *Pseudomonas putida* (17), and *Escherichia coli* (18), provides a sustainable alternative. Living cells act as both carriers and catalysts, offering biocompatibility, ease of scaling, and enhanced enzyme protection.

Among the various microbial display systems, Gram-positive bacteria such as *Bacillus subtilis* stand out for their robust cell walls and ability to present proteins on their surface. One of the most promising techniques for anchoring proteins to Gram-positive bacterial surfaces is sortase-mediated immobilization. This method uses sortase enzymes to covalently attach proteins to the bacterial cell wall. Proteins targeted for sortase anchoring typically include an N-terminal signal peptide for secretion and a C-terminal cell wall sorting signal, which comprises a recognition motif (e.g., leucine-proline-aspartate-threonine-serine or LPDTS in *B. subtilis*), a hydrophobic region, and a charged tail (19, 20). Sortase enzymes, such as YhcS in *B. subtilis*, cleave the sorting motif and covalently link the protein to the peptidoglycan layer, ensuring stable immobilization (21). The covalent nature of the attachment via sortase also ensures that the enzyme remains firmly bound during reaction cycles, allowing for repeated use without significant loss of activity. This is crucial for industrial applications where enzyme reusability is a key economic factor (22). Despite significant progress in enzyme immobilization, many existing techniques lack the simplicity, scalability, and cost-efficiency required for industrial adoption. Our study addresses these gaps by employing sortase-mediated immobilization to develop a novel whole-cell biocatalyst. Specifically, we successfully displayed CALB on the surface of *B. subtilis* cells using YhcS sortase and the sorting signal derived from its natural substrate, YhcR. This approach integrates the benefits of enzyme immobilization with the versatility of living cells, creating a thermostable, reusable, and environmentally sustainable biocatalyst.

By advancing sortase-based enzyme immobilization technology, this study paves the way for scalable and cost-effective solutions in biocatalysis, with potential applications across food processing, pharmaceutical production, and biodiesel synthesis. The use of *B. subtilis* as a host further enhances the practicality of this method, offering a robust, safe, and easily manipulated platform for industrial bioprocessing.

## MATERIALS AND METHODS

### Bacterial strains, media and growth conditions

Fish oil derived from menhaden (containing 10%–15% eicosapentaenoic acid [EPA] and 8%–15% docosahexaenoic acid [DHA]), commercial standards of EPA and DHA (used for hydrolysis reaction quantification), and 4-Nitrophenyl butyrate (*p*-NPB) were purchased from Sigma-Aldrich. The bacterial strains and plasmids utilized in this study are detailed in Table 1. *Escherichia coli DH5α* and *Bacillus subtilis WASD* served as the host strains for general cloning and lipase surface display experiments, respectively.

**TABLE 1:** Bacterial strains and plasmids

Plasmids and strains		Relevant properties References
Plasmids		
pHY300-PLK	*B. subtilis* protein expression vector; Apr, Tcr	NIGEB collection
pSpA-YhcS	Recombinant pHY plasmid containing spa gene and *B. subtilis* sortase (YhcS)	Previous study
pCALB-YhcS	Recombinant pHY plasmid containing lipase gene and *B. subtilis* sortase (YhcS)	This study
Strains		
*E. coli i*(DH5α)	F- endA1 glnV44 thi-1 recA1 relA1 gyrA96 deoR nupG Φ80dlacZΔM15 Δ(lacZYA-argF)U169, hsdR17(rK- mK+), λ–	Invitrogen
*B. subtilis*(WASD)	trpC wprA::kan sigD::cat	NIGEB collection
*Bs*.pHY	*B. subtilis* (WASD) transformed with pHY300-PLK	Previous study
*Bs-CALB*	*B. subtilis* (WASD) transformed with pCALB-YhcS	This study

*E. coli* cells were cultured at 37°C in Luria-Bertani (LB) medium, which consisted of 1% (wt/vol) tryptone, 0.5% (wt/vol) yeast extract, and 1% (wt/vol) NaCl. The medium was supplemented with 50 µg/mL ampicillin for antibiotic selection. For protein production, *B. subtilis* cells were grown in super-rich medium (SRM) containing Bacto tryptose (25 g/L), yeast extract (20 g/L), dipotassium phosphate (3 g/L), and glucose (4.5 g/L) at pH 7.5. Cultures were supplemented with 25 µg/mL tetracycline and incubated at 30°C for 14 hours before harvesting for downstream analyses.

### Construction of plasmids and expression

Primers LIPF1 and LIPR1, containing *XhoI* and *BamHI* restriction sites, respectively (Table 2), were used to amplify the CALB gene. The CALB gene, previously cloned into the pET41a vector (22), served as the template for the PCR. The amplified CALB fragment was digested with *XhoI* and *BamHI* and subsequently ligated into the corresponding sites of the plasmid pSpA-YhcS. This plasmid was originally designed for protein A surface display in a prior study, where protein A was successfully displayed on the surface of *B. subtilis*.

**TABLE 2:** Oligonucleotide primer sequences used for CALB amplification

Primer name	Sequence
LIPF1 (forward)	5' GGGGCTCGAGCTGCGAGCGGTAGCGATCC 3'
LIPR1 (reverse)	5' GGGG*GGATCC*CGGGGTCACAATACCGCTACA 3'

In this study, the SpA gene was excised from the pSpA-YhcS plasmid using *XhoI* and *BamHI* restriction enzymes, and the CALB gene was inserted into the backbone of the construct. The resulting recombinant plasmid was designated pCALB-YhcS. Plasmid pHY300 with no insert was used as a negative control.

The above plasmids were used to transform competent *E. coli* DH5α cells via the thermal shock method (23), using the standard calcium chloride protocol. Transformed colonies were screened in LB medium containing 50 µg/mL ampicillin. After confirming the sequences of the fusion protein-encoding regions through DNA sequencing, *B. subtilis* WASD cells were transformed with the recombinant plasmid pCALB-YhcS and a control plasmid, pHY300PLK (lacking both the CALB gene and sortase). pHY300PLK, a shuttle vector compatible with both *E. coli* and *Bacillus subtilis*, equipped with ampicillin and tetracycline resistance markers. Transformation was performed using Spizizen’s method (24), and the natural transformation approach, wherein *B. subtilis* cultures were subjected to conditions that induced DNA uptake from the environment (25).

For surface expression of the CALB gene in *B. subtilis*, a single colony containing the recombinant plasmid was inoculated into 5 mL of LB medium supplemented with tetracycline and incubated at 37°C with shaking at 180 rpm. After 16 hours, 0.5 mL of the primary culture was transferred into 50 mL of SRM and incubated for 6 hours at 37°C with shaking at 100 rpm. Subsequently, half of the initial amount (25 µL) of tetracycline was added to enhance the induction of expression, and the culture was incubated for an additional 16 hours under the same conditions. The bacterial cells were then harvested by centrifugation for further analysis. CALB was expressed under the control of the tetracycline promoter (*tetR*), which in this vector functions as a constitutive promoter in *B. subtilis*.

### Preparation of cell wall and protoplast fractions of the surface-engineered *B. subtilis* and analysis by SDS-PAGE

A 50 mL overnight culture of *B. subtilis* was harvested by centrifugation at 4,500 rpm for 20 minutes. The resulting pellet was washed and resuspended in 800 µL of protoplasmic buffer (20% sucrose, 50 mM Tris, pH 7.5, 15 mM MgCl2, 0.2 mg/mL lysozyme, pH 7.6) to isolate the cell wall fraction. The samples were incubated at 37°C for 90 minutes. Following incubation, the suspension was centrifuged at 12,000 rpm for 20 minutes.

The pellet, containing the cell wall fraction, was dissolved in phosphate buffer for subsequent applications. Both the cell wall and protoplast fractions were subjected to further analyses, including SDS-PAGE.

### Measurement of the whole-cell enzyme activity assay

Enzyme activity was assessed by analyzing optical density (OD) at 410 nm, using *p*-nitrophenyl butyrate (*p*-NPB) as the substrate. The reaction was monitored over a period of 2 minutes, with measurements taken every 15 seconds. For this assay, *B. subtilis* cells harboring either the control plasmid (Bs-pHY) or the expression plasmid (Bs-CALB) were cultured, and gene expression was induced as described in the previous sections. After induction, the cells were harvested by centrifugation, and the resulting pellets were resuspended in 10 mM sodium phosphate buffer (pH 8.0). The optical density of the cell suspensions at 600 nm (OD_600_) was measured, and the concentrations were adjusted to ensure equal cell densities across samples.

To determine enzyme activity in whole-cell lysates, equalized cell suspensions of Bs.pHY (as a negative control) and Bs-CALB were mixed with 10 µL of *p*-NPB in 10 mM sodium phosphate buffer (pH 8.0) at a reaction temperature of 25°C. The release of *p*-nitrophenol, a product of *p*-NPB hydrolysis, was monitored by UV spectrophotometry at 410 nm. One unit of enzyme activity was defined as the amount of enzyme required to release 1 µmol of *p*-NPB per minute under the assay conditions. All measurements were performed in triplicate to ensure reproducibility, and mean values were reported along with standard deviations to indicate variability. Bs.pHY was rigorously included in all functional assays and processed in parallel with Bs-CALB under identical conditions.

### Assessment of thermal stability and denaturation profile of Bs-CALB

The thermal stability of *B. subtilis* cells displaying *Candida antarctica* Lipase B (Bs-CALB) was evaluated by assessing enzyme activity at four temperatures (37°C, 40°C, 45°C, and 50°C) in 10 mM sodium phosphate buffer (pH 8.0) for 0–8 hours. Bs-CALB cells (OD_600_ = 1.0, equivalent to 0.3 g/L dry cell weight [DCW]) were incubated, and aliquots were withdrawn at each time point to measure residual lipase activity using the p-nitrophenyl butyrate (p-NPB) assay (0.4 mM p-NPB, 37°C, pH 8.0) by monitoring p-nitrophenol release at 410 nm, as described before. Free CALB was tested under identical conditions for comparison. The T₅₀ (temperature at which 50% activity is retained after 8 hours) was calculated by interpolating the residual activity data at 37°C, 40°C, 45°C, and 50°C using a sigmoidal fit. All experiments were performed in triplicate, and results are reported as means ± standard deviations.

### Determination of pH profile of Bs-CALB and free recombinant CALB

The effect of pH on enzyme activity was evaluated to establish the pH profile of both Bs-CALB (surface-displayed CALB) and free recombinant CALB. Enzyme activity assays were performed in 10 mM sodium phosphate buffers with pH values ranging from 4.5 to 9.5. Each reaction was carried out under standard assay conditions, as described previously, using 10 µL of *p*-nitrophenyl butyrate (*p*-NPB) as the substrate.

The relative activity of the enzymes at each pH was determined by comparing the activity to that observed under optimal pH conditions (pH 8.0 for Bs-CALB). All measurements were conducted in triplicate, and mean values were reported alongside standard deviations to ensure accuracy and reproducibility.

### Evaluation of continuous lipase activity of Bs-CALB over 24 hours

The temporal stability of surface-displayed CALB (Bs-CALB) was rigorously assessed by monitoring enzyme activity at regular intervals. Specifically, enzyme activity measurements were taken every 2 hours across 24 hours, under pre-optimized catalytic conditions of 37°C and pH 8.0. Each sample underwent analysis via the standard assay protocol, with relative activity quantified as a percentage of the initial activity observed at time zero.

To ensure the statistical reliability and robustness of the data, all experiments were conducted in triplicate. Results are presented as mean values accompanied by standard deviations.

### Assessment of the reusability of Bs-CALB

To evaluate the reusability of *B. subtilis* cells displaying CALB (Bs-CALB), reuse cycle experiments were conducted as follows. Each cycle consisted of a 20 minute reaction at 37°C in 10 mM sodium phosphate buffer (pH 8.0) containing 0.4 mM p-nitrophenyl butyrate (p-NPB) as the substrate. The reaction was initiated by resuspending Bs-CALB cells (OD_600_ = 1.0, equivalent to 0.3 g/L DCW) in 1 mL of reaction mixture. After each cycle, cells were recovered by centrifugation at 4,500 rpm (approximately 3,000 × *g*) for 10 minutes at 4°C, washed three times with 1 mL of 10 mM sodium phosphate buffer (pH 8.0) to remove residual substrate and product, and immediately resuspended in a fresh reaction mixture for the next cycle. The interval between cycles was approximately 15 minutes, accounting for centrifugation and washing steps. Lipase activity was measured after each cycle by monitoring p-nitrophenol release at 410 nm, as described before. Cell density (OD_600_) was measured before and after each cycle to assess biomass stability, and supernatant samples were tested for lipase activity to evaluate enzyme leaching. All experiments were conducted in triplicate, and results are reported as means ± standard deviations.

### Assessment of long-term storage stability of Bs.CALB cells

The storage stability of surface-displayed CALB (Bs.CALB) on *B. subtilis* cells was evaluated over an extended period of 8 weeks. Enzyme activity was measured weekly under previously established optimal catalytic conditions (37°C and pH 8.0) to monitor any changes in performance. Recombinant *B. subtilis* cells were stored at 4°C in 10 mM sodium phosphate buffer (pH 8.0) without any additional stabilizing agents. At each time point, aliquots were withdrawn, and enzyme activity was assessed using the standard assay with *p*-nitrophenyl butyrate (*p*-NPB). Residual activity was expressed as a percentage of the initial activity measured at week 0.

All experiments were conducted in triplicate to ensure statistical reliability, and results were reported as mean values with standard deviations. This approach provided a robust evaluation of the long-term stability of Bs.CALB cells, highlighting their suitability for industrial and biotechnological applications.

### Fourier-transform infrared analysis of functional groups in Bs-CALB cells

Fourier-transform infrared (FT-IR) spectra were recorded using a Bruker-Vector22 spectrometer (Bruker, Billerica, MA, USA) within the wavelength range of 4,000–400 cm⁻¹ to analyze the functional groups present in free recombinant CALB, free untreated *B. subtilis* cells, and Bs.CALB cells.

### Evaluation of enzymatic activity and selectivity in fish oil hydrolysis using Bs-CALB cells

The enzymatic activity and selectivity of *Bacillus subtilis* cells displaying CALB (Bs-CALB) were evaluated in the hydrolysis of fish oil in an emulsified system. To prepare the reaction, 5 mL of Bs.CALB cell suspension in 10 mM sodium phosphate buffer (pH 8.0) was mixed with 1.5 mL of deionized water containing 170 µL of fish oil and 3 mg of bovine serum albumin as an emulsifier. The mixture was stirred at two reaction temperatures, 25°C and 40°C, to assess the effect of temperature on the hydrolytic activity and substrate selectivity of Bs-CALB.

At predetermined intervals, 500 µL of the samples was collected, and fatty acids released during hydrolysis were extracted using 1 mL of cyclohexane. The concentrations of EPA and DHA in the extracted samples were quantified using high-performance liquid chromatography (HPLC). HPLC analysis was performed on an Agilent-1260A system equipped with a Eurospher 100-5 C18 column (250 mm × 4.6 mm) using a mobile phase comprising 67% acetonitrile and 33% water containing 0.5% acetic acid, with a flow rate of 0.7 mL/min. Detection was carried out at 210 nm, with retention times of 20 minutes for EPA and 25 minutes for DHA. Standard curves of pure EPA and DHA were used to calculate the fatty acid concentrations. The selectivity of the hydrolysis reaction was defined as the ratio of EPA to DHA concentrations in the reaction products. All experiments were performed in duplicate, and results were reported as mean values with standard deviations to ensure accuracy and reproducibility.

## RESULTS AND DISCUSSION

### Construction of recombinant plasmids

**Fig 1:**
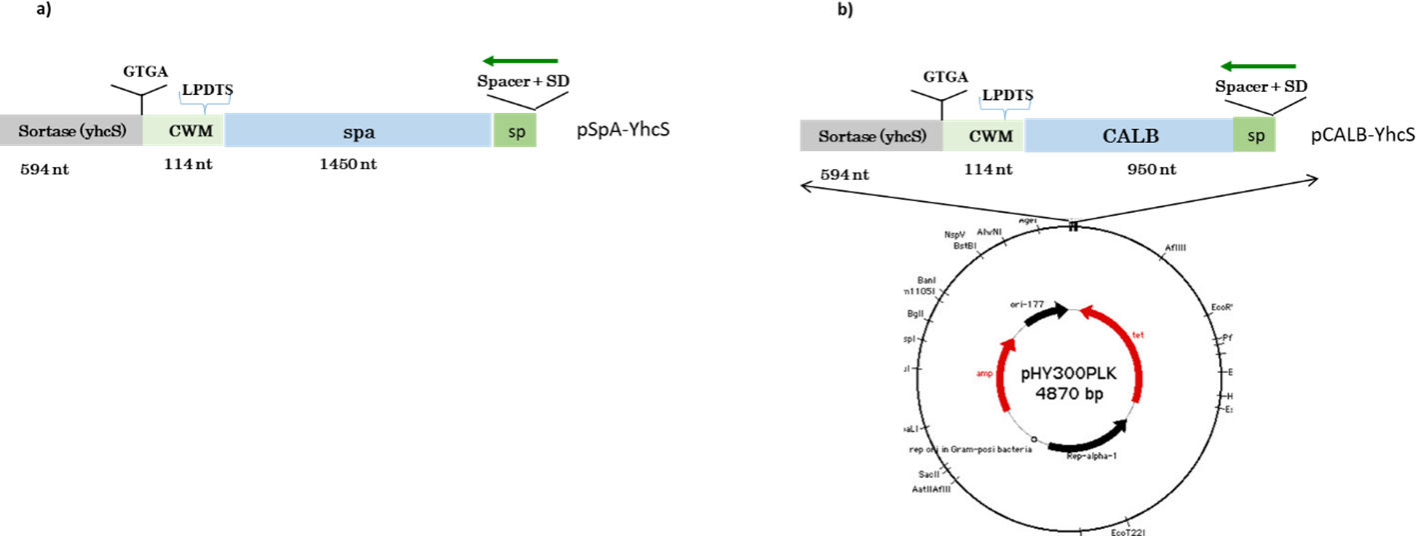
Schematic diagram of plasmid constructs and surface display fusion proteins. (**a**) The pSpA-YhcS plasmid encoding the spa gene from *S. aureus*, fused to the native *B. subtilis* cell wall sorting motif (CWM) derived from YhcR, and co-expressed with the yhcS sortase gene under the constitutive tetR promoter in the pHY300PLK backbone. (**b**) The spa gene was replaced with the CALB gene, yielding plasmid pCALB-YhcS, which preserves the co-expression design and transcriptional overlap between CALB and yhcS to facilitate sortase-mediated immobilization of CALB on the surface of *B. subtilis* WASD.

In this study, recombinant plasmids for the surface display of CALB on *Bacillus subtilis* were successfully constructed, as illustrated in Fig. 1. The target gene encoding CALB was positioned downstream of the tetracycline resistance gene in the plasmid pHY300, ensuring constitutive expression under the control of its native promoter. The fusion plasmid, designated pCALB-YhcS, was engineered to include a Shine-Dalgarno (SD) sequence for efficient translation initiation, the CALB coding region for lipase activity, and a cell wall motif (CWM) containing the LPDTS recognition sequence. This motif enabled sortase-mediated anchoring of CALB to the bacterial cell wall. Additionally, the plasmid encoded the sortase enzyme YhcS, native to *B. subtilis*, which facilitated the covalent attachment of CALB to the cell wall via the LPDTS motif.

The design of pCALB-YhcS ensured efficient co-expression of CALB and YhcS, promoting robust immobilization of the enzyme on the *B. subtilis* cell surface. This dual-component system mimicked the natural protein sorting mechanism in *B. subtilis* and was pivotal for creating a functional whole-cell biocatalyst (Fig. 1).

The combination of *B. subtilis* and sortase-mediated immobilization offers several advantages. *B. subtilis* is a well-established model organism with robust cell wall properties, making it an ideal host for protein display. The sortase-mediated system ensures that target proteins are stably anchored to the cell wall, maintaining their functional integrity and accessibility. This technique enhances the stability and reusability of immobilized enzymes while simplifying the production process by eliminating the need for additional purification or chemical cross-linking steps. Sortase-mediated protein immobilization has been extensively used *in vitro* for protein labeling, oligomerization, circularization, and immobilization (26). It allows for site-specific and efficient coupling under physiological buffer conditions, preserving protein function. In our study, this approach was successfully applied for lipase immobilization on the bacterial cell surface.

Previous studies have also utilized sortase for enzyme immobilization on non-living surfaces. For example, Parthasarathy et al. (27) immobilized green fluorescent protein on polystyrene beads (27), while Hata et al. (28) immobilized an α-amylase on glycine-conjugated polystyrene nanoparticles, examining their activity, stability, and reusability (28).

Moosavi et al. (29) immobilized CALB on graphene oxide using oriented and random immobilization approaches (29). Although effective, these techniques are often sophisticated and require multiple steps, including surface preparation, enzyme purification, and cross-linking. In contrast, our approach, which utilizes the bacterial surface as an immobilization medium, is more straightforward and cost-effective.

The engineered *B. subtilis* strain (Bs-CALB) adheres to internationally accepted biosafety frameworks governing genetically modified microorganisms. A critical factor in regulatory compliance is the selection of the host organism, as *B. subtilis* is designated as Generally Recognized as Safe (GRAS) by the U.S. Food and Drug Administration (30) and is listed under the Qualified Presumption of Safety (QPS) status by the European Food Safety Authority (31). These designations are based on the organism’s long history of industrial use and its non-pathogenic profile (31, 32).

Operational containment further enhances biosafety. Bs-CALB is cultured in closed bioreactor systems compliant with OECD containment Level 1 standards, which effectively prevent environmental release. Additionally, the final products, such as omega-3 fatty acids, undergo rigorous downstream purification processes that remove residual cellular components, ensuring product safety and purity. Notably, the Bs-CALB whole-cell system is compatible with regulatory provisions for carrier-bound immobilized enzymes (33), wherein the bacterial carrier does not directly contact the final product, thereby mitigating safety concerns.

### Validation of cell wall-anchored CALB expression on *Bacillus subtilis* surface via SDS-PAGE

**Fig 2:**
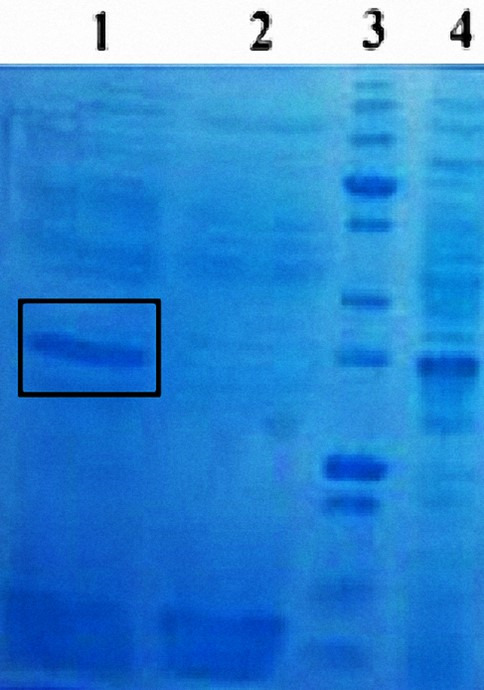
SDS-PAGE analysis of CALB expression and localization. This figure presents the SDS-PAGE analysis demonstrating the expression and localization of CALB. Lane 1 shows the 33 kDa band corresponding to surface-displayed CALB on Bs-CALB cells at 16 hours post-induction, following lysozyme treatment. Lane 2 represents the Bs.pHY control sample with no CALB expression, which shows no 33 kDa band in the cell wall fraction, confirming the absence of CALB. The molecular weight markers in Lane 3 provide a reference. Lane 4 confirms consistent CALB expression at 16 hours in *Escherichia coli*. These results establish the functionality of the sortase-mediated immobilization system and its capability to specifically and efficiently localize CALB on the *B. subtilis* cell wall. Samples (20 µg protein/lane) were prepared after 16 hours of growth in LB medium with tetracycline (25 µg/mL) at 37°C, with three biological replicates.

The successful immobilization of CALB on the *B. subtilis* cell surface was confirmed through SDS-PAGE analysis of protein fractions isolated from recombinant cells (Bs.CALB) and control cells (Bs.pHY). To prevent contamination of cell wall proteins with cytoplasmic or protoplasmic proteins, the cells were treated with lysozyme. This enzymatic shaving process selectively removed cell wall-anchored proteins while preserving protoplast integrity, enabling precise comparative analysis. Protein fractions derived from both the cell wall and protoplast of Bs-CALB and Bs.pHY cells were subjected to SDS-PAGE. Figure 2 shows a distinct band at approximately 33 kDa in Bs-CALB samples, corresponding to the molecular weight of CALB (34), which is absent in the empty vector control, confirming successful expression and sortase-mediated surface display. The experiment was conducted using a 12% polyacrylamide gel with Coomassie Blue staining, with samples normalized to equal protein loading (20 µg per lane) and run in triplicate to ensure reproducibility. Minor background bands, typical in whole-cell lysates, were observed but did not obscure the CALB band. Additionally, the absence of a 33 kDa band in the protoplast fractions of Bs-CALB samples further validated that CALB was exclusively anchored to the cell wall and not retained intracellularly. These results demonstrate the efficiency and specificity of the sortase-mediated system in immobilizing CALB on the *B. subtilis* cell surface.

### Validation and quantification of CALB immobilization efficiency

**Fig 3:**
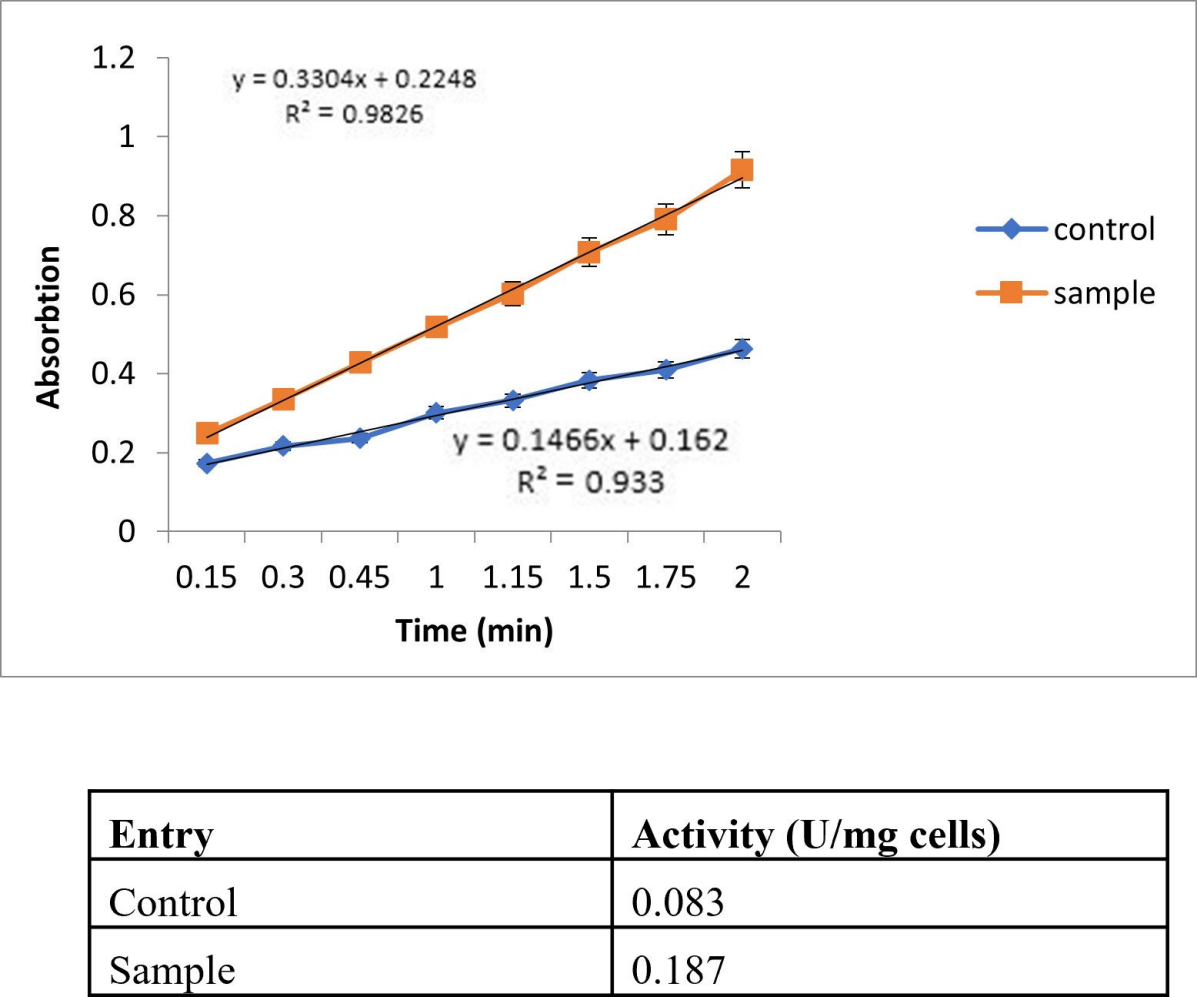
Lipase activity of recombinant *Bacillus subtilis* cells displaying CALB (Bs-CALB) and negative control (Bs.pHY).

To validate the efficiency of sortase-mediated immobilization of CALB on the *Bacillus subtilis* cell wall, we employed SDS-PAGE analysis of cell wall fractions, which revealed a distinct band at approximately 33 kDa in Bs-CALB cells, consistent with CALB’s molecular weight, but not in control cells (Bs.pHY) (Fig. 2). This confirms the presence of CALB on the cell surface. However, precise quantification via densitometry was not feasible due to the unavailability of an anti-CALB antibody for specific detection in Western blot analysis, which is typically required for accurate band intensity measurement. Similarly, the bicinchoninic acid (BCA) assay was not used, as it measures total protein content and cannot distinguish surface-displayed CALB from other cell wall-associated proteins in *B. subtilis*, potentially leading to overestimation (35). To address this, we quantified CALB immobilization yield using the lipase activity data from the p-nitrophenyl butyrate (p-NPB) assay (0.187 ± 0.015 U/mg DCW, Fig. 3) and the reported specific activity of purified CALB (10 U/mg CALB) (36). This yielded an estimated CALB immobilization of 18.7 ± 1.5 µg/g DCW, equivalent to 5.61 ± 0.45 µg/mL at OD_600_ = 1.0 (0.3 g/L DCW, 8 × 10^8^ cells/mL), or 7.01 ± 0.56 pg/cell (approximately 1.28 × 10^5^ CALB molecules/cell, based on a 33 kDa molecular weight). These estimates, derived from triplicate measurements, provide a quantitative measure of immobilization efficiency.

The sortase-mediated covalent anchoring was further validated by the absence of lipase activity in the supernatant of Bs-CALB cultures, indicating that CALB remained bound to the cell wall, unlike non-covalently immobilized enzymes that may leach. Compared to other immobilization methods, such as adsorption on hydrophobic supports, which often result in enzyme leaching (e.g., 20%–30% activity loss after five cycles) (36), our system’s performance underscores the efficacy of sortase-mediated anchoring. While direct protein quantification via densitometry or BCA would enhance precision, the activity-based estimation and functional assays confirm efficient CALB immobilization. Future studies will explore fluorescence-based tagging or proteomic analysis to directly quantify surface-displayed CALB, building on the foundation established here (35).

### Evaluation of whole-cell lipase activity in Bs-CALB cells

The lipase activity of *Bacillus subtilis* cells displaying CALB (Bs-CALB) was rigorously evaluated by quantifying the release of *p*-nitrophenol from the hydrolysis of *p*-NPB. This enzymatic assay served as a robust method to confirm the functional expression of CALB on the surface of recombinant Bs-CALB cells. Recombinant Bs-CALB cells exhibited significantly higher lipase activity compared to control Bs.pHY cells, which lack the CALB gene. This marked increase in activity observed in Bs-CALB cells confirmed the successful surface expression and enzymatic functionality of CALB. The assay results, illustrated in Fig. 3, demonstrate the substantial catalytic advantage provided by surface-displayed CALB. The higher release of *p*-nitrophenol in Bs-CALB samples relative to controls (Bs.pHY) underscores the efficiency of the sortase-mediated system in immobilizing and activating CALB on the *Bacillus subtilis* cell surface, confirming that catalysis is exclusively due to surface-displayed CALB.

Notably, the Bs-CALB cells displayed a consistent and robust lipase activity over the duration of the assay, further highlighting the efficiency of the surface-displayed enzyme. This enhanced activity can be attributed to the strategic positioning of CALB on the bacterial surface, which facilitates optimal substrate access and turnover, thereby enhancing catalytic efficiency. Therefore, the sortase-mediated immobilization technique ensures the stable anchoring of the enzyme while preserving its catalytic properties, making it a valuable approach for whole-cell biocatalysis.

The graph illustrates the increased absorption at 410 nm, corresponding to the release of *p*-nitrophenol from *p*-NPB by surface-displayed CALB. The linear slope of 0.33 observed in Bs.CALB cells confirms active lipase expression on the cell surface. In contrast, the control cells (Bs.pHY) exhibit lower activity, indicating the specificity of CALB expression and functionality in recombinant cells.

### Estimation of CALB expression yield on the *B. subtilis* surface

To quantify the amount of CALB displayed on the surface of *Bacillus subtilis* (Bs-CALB), we estimated the enzyme yield using the lipase activity data from the p-nitrophenyl butyrate (p-NPB) assay (0.187 ± 0.015 U/mg DCW, Fig. 3) and the reported specific activity of purified CALB (10 U/mg CALB) (36). By dividing the whole-cell activity by the specific activity of purified CALB, we calculated an estimated CALB yield of 18.7 ± 1.5 µg per gram of dry biomass. For a culture at OD_600_ = 1.0, equivalent to approximately 0.3 g/L DCW and 8 × 10^8^ cells/mL, this corresponds to 5.61 ± 0.45 µg CALB per mL of culture, or approximately 7.01 ± 0.56 pg CALB per cell (equivalent to 1.28 × 10^5^ CALB molecules per cell, based on a molecular weight of 33 kDa). These estimates were derived from triplicate measurements, with standard deviations reflecting variability in the activity assays.

This estimated yield facilitates comparison with other CALB immobilization systems. For instance, Novozym 435, a commercially available immobilized CALB on acrylic resin, typically contains 10–20 mg CALB per gram of support, with a specific activity of approximately 0.01–0.02 U/µg (8, 37). Although the CALB loading in Bs-CALB (18.7 µg/g DCW) is lower, the specific activity of the enzyme (0.01 U/µg CALB, calculated as 0.187 U/mg DCW ÷ 18.7 µg CALB/g DCW) is comparable, suggesting efficient enzyme orientation and accessibility on the cell surface due to sortase-mediated immobilization.

Furthermore, the specific activity per biomass of Bs-CALB (0.187 ± 0.015 U/mg DCW) is competitive with other microbial display systems, such as CALB displayed on *Pichia pastoris* (~0.15 U/mg DCW) moura (38), highlighting the efficacy of our whole-cell biocatalyst. The use of living cells as a support eliminates the need for costly purification and chemical cross-linking, enhancing the cost-effectiveness of this approach. We acknowledge that direct protein quantification (e.g., via Western blot) was not feasible due to the unavailability of an anti-CALB antibody. Future studies will explore alternative methods, such as fluorescence-based tagging or proteomic analysis, to validate these estimates.

### Recent advances in enzyme surface display in Gram-positive bacteria

Recent advancements in enzyme surface display technologies for Gram-positive bacteria, particularly *Bacillus subtilis* and *Lactococcus lactis*, have leveraged genetic engineering tools to enhance expression stability and industrial applicability. CRISPR-Cas9 has been employed to integrate genes encoding enzymes like β-galactosidase into the *B. subtilis* chromosome, achieving stable expression comparable to multi-copy plasmids without antibiotic selection (39). For instance, five copies of the *ganA* gene were integrated into the *B. subtilis* chromosome using CRISPR-Cas9, resulting in β-galactosidase activity sustained over 40 generations, unlike plasmid-based systems that lost activity under non-selective conditions (39). Similarly, CRISPR-Cas9 has been used to delete genes encoding extracellular proteases in *B. subtilis*, such as in the genome-reduced strain IIG-Bs-27-39, which lacks 21.6% of the genome and exhibits enhanced secretion of protease-sensitive proteins (40). In *L. lactis*, the nisin-controlled expression (NICE) system, combined with chromosomal integration, has enabled stable production of vaccine antigens and therapeutic proteins with minimal proteolysis due to the absence of extracellular proteases (41). Sortase-mediated anchoring, as used in our study, remains a cornerstone of surface display, but recent work has optimized this approach by integrating sortase recognition motifs (e.g., LPDTS) into chromosomally expressed fusion proteins, ensuring covalent attachment and enhanced stability (42). These methods reduce dependence on antibiotics and mitigate plasmid instability, making them ideal for industrial bioprocessing (35). In our study, a plasmid-based vector was employed for rapid functional testing of CALB surface display on *B. subtilis*, achieving a specific activity per biomass of 0.187 ± 0.015 U/mg DCW (Fig. 3). While effective for initial validation, we acknowledge that plasmid systems may exhibit instability in large-scale fermentations (35). Future work will explore CRISPR-Cas9-mediated chromosomal integration of the CALB gene, potentially targeting loci like *amyE* for high expression, as demonstrated in recent studies (39), and deletion of protease genes to further enhance CALB yield and stability (40). These advancements will build on the foundation established in this study, aligning our approach with state-of-the-art enzyme display technologies for sustainable and cost-effective biocatalysis.

### Thermostability, pH, and solvent stability of surface-displayed CALB

**Fig 4:**
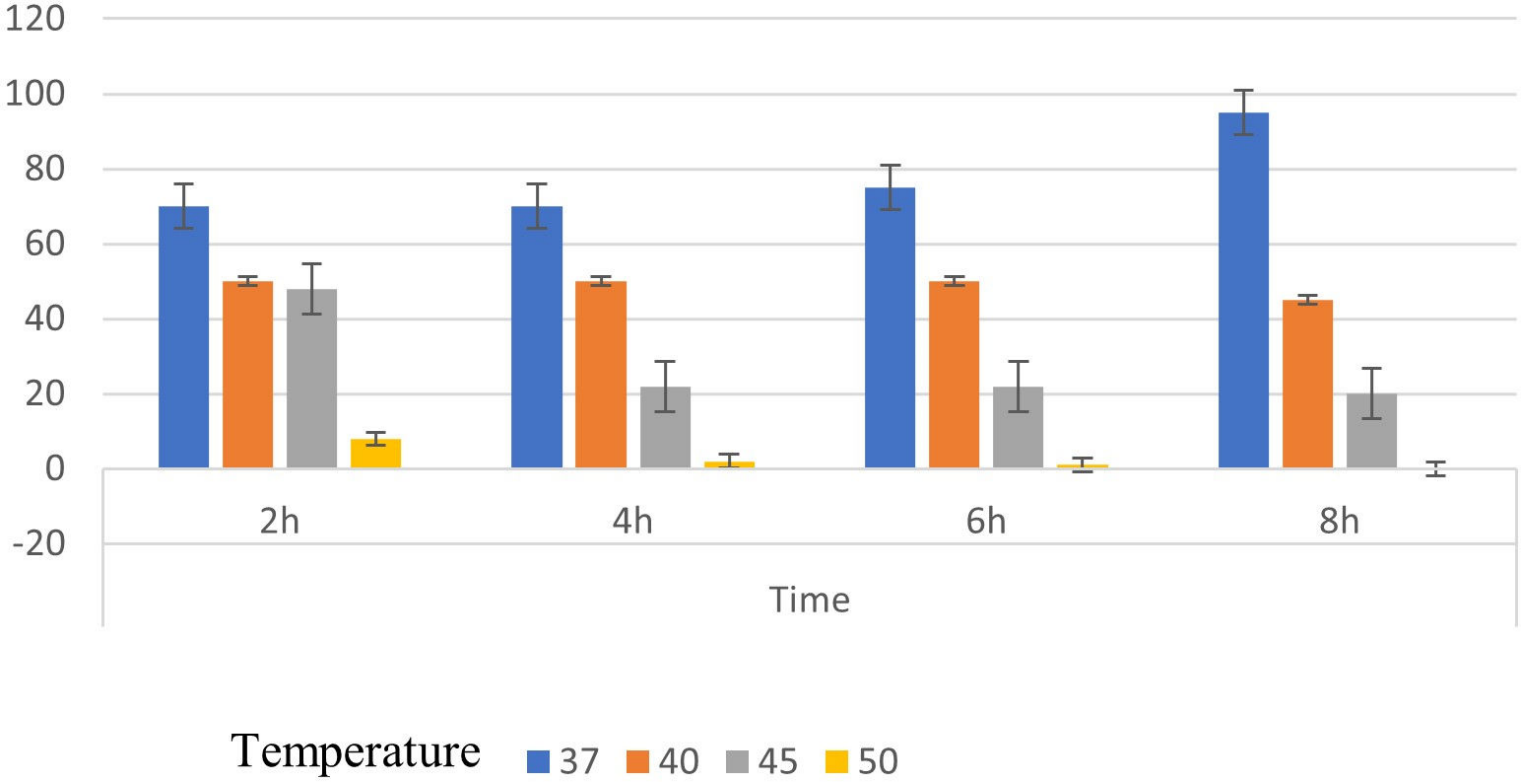
Thermal stability analysis of surface-displayed CALB on *Bacillus subtilis*. The graph illustrates the thermal stability of surface-displayed CALB (Bs.CALB) at various temperatures (37°C, 40°C, 45°C, and 50°C) over an 8-hour incubation period. Different colored columns represent residual enzymatic activity at each temperature, expressed as a percentage of the initial activity.

The thermostability of *Bacillus subtilis* cells displaying CALB (Bs-CALB) was evaluated by measuring residual enzymatic activity over an 8-hour incubation period at four temperatures (37°C, 40°C, 45°C, and 50°C) in 10 mM sodium phosphate buffer (pH 8.0). Bs-CALB exhibited optimal activity at 37°C, retaining 93.7% ± 1.8% of its initial activity (0.187 ± 0.015 U/mg DCW) after 8 hours, demonstrating remarkable stability suitable for biologically relevant applications. At 40°C, the enzyme retained 50.3% ± 2.4% activity, significantly outperforming free CALB, which retained only 10.2% ± 1.5% activity under the same conditions. At higher temperatures, activity declined more rapidly, with 23.1% ± 3.1% at 45°C and 5.6% ± 1.2% at 50°C (Fig. 4). At higher temperatures (40°C–50°C), although the activity declined, Bs-CALB retained a measurable portion of its initial activity compared to free CALB, which typically loses nearly all activity at these temperatures, indicating resilience conferred by surface immobilization.

To quantify thermal denaturation, the T₅₀ (temperature at which 50% activity is retained after 8 hours) was calculated by interpolating the activity data using a sigmoidal fit, yielding a T₅₀ of approximately 40.2°C for Bs-CALB, compared to 36.5°C for free CALB. This enhanced thermal stability is attributed to the sortase-mediated covalent anchoring, which stabilizes the enzyme’s active site and reduces denaturation under thermal stress (43).

The increase in lipase activity during the first 8 hours can be explained by the “lag phase” of enzyme kinetics. During this initial period, the enzyme may undergo conformational changes that enhance its ability to effectively interact with substrates. This phase allows the enzyme to optimize its active site for substrate binding, leading to increased catalytic efficiency as the reaction progresses (44). In terms of temperature stability, Bs-CALB exhibited excellent performance. At 37°C, the enzyme maintained its activity over an extended period, making it suitable for processes that operate at this temperature.

This stabilization effect demonstrates the effectiveness of the sortase-mediated immobilization approach in protecting the enzyme’s functional integrity under thermal stress. By enabling site-specific attachment to a stable matrix, this method reduces enzyme denaturation and aggregation often observed in free enzymes under challenging conditions. Selecting 37°C as the operational temperature for subsequent experiments maximized enzyme activity and ensured long-term stability, highlighting its industrial potential. These findings underscore the versatility of this immobilization strategy in enhancing enzyme performance across varying conditions (22, 45).

At 37°C, Bs-CALB exhibited high thermal stability, retaining 93.7% ± 1.8% of its initial activity after 8 hours (Fig. 4). An apparent slight increase in residual activity at early time points may be observed, which is likely attributable to transient enzyme activation or minor assay variability. Immobilized lipases, including Bs-CALB, can undergo conformational stabilization at optimal temperatures due to interactions with the cell surface, temporarily enhancing activity, as reported for lipases on hydrophobic supports (46). Additionally, the p-nitrophenyl butyrate (p-NPB) assay may introduce slight variability (e.g., substrate solubility or cell settling), contributing to minor fluctuations within the experimental error (±1.8%). These factors do not detract from the overall finding that Bs-CALB maintains excellent stability at 37°C, significantly outperforming free CALB (10.2 ± 1.5% at 40°C), and supports its suitability for industrial applications (43).

**Fig 5:**
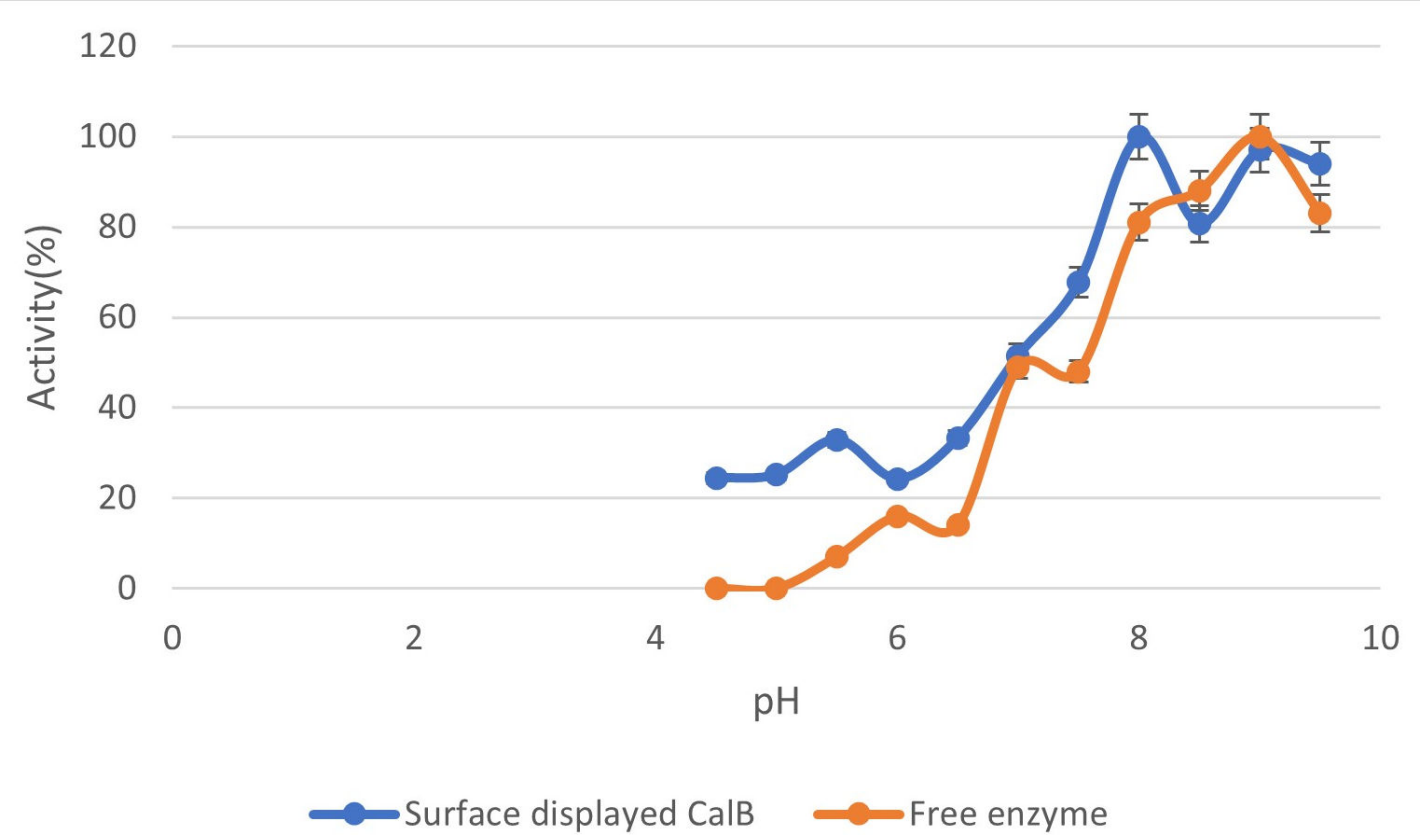
pH stability of Bs-CALB and free CALB. Residual activity (% of maximum at pH 8.0) of Bs-CALB (blue) and free CALB (orange) was measured after 2 hours at 37°C in 10 mM sodium phosphate buffers (pH 4.5–9.5) using the p-nitrophenyl butyrate (p-NPB) assay. Data points represent mean activity from three biological replicates, with error bars indicating standard deviations. The scatter plot with connected lines illustrates the pH-dependent activity trend, with Bs-CALB showing optimal stability at pH 8.0. Control cells (Bs.pHY) exhibited negligible lipase activity (<0.05 U/mg) under all tested pH conditions (data not shown).

The pH stability of Bs-CALB was assessed across pH 4.5–9.5 in 10 mM sodium phosphate buffers at 37°C for 2 hours. Bs-CALB achieved maximum activity at pH 8.0, retaining 94.2% ± 1.6% of its initial activity, with 80.4% ± 1.8% at pH 6.0, 68.3% ± 2.1% at pH 4.5, and 72.5% ± 2.4% at pH 9.5 (Fig. 5). This broad pH stability, compared to free CALB’s sharper decline at non-optimal pH (e.g., 50.1% ± 2.8% at pH 4.5), reflects the structural reinforcement provided by immobilization, which minimizes conformational changes under suboptimal conditions (47). The slight shift in optimal pH (8.0 for Bs-CALB vs. 9.0 for free CALB) likely results from altered surface charge due to cell wall anchoring (48).

The slight difference between 94.2% (2 hours, pH stability) and 93.7% (8 hours, thermal stability) reflects the longer incubation time in the thermal assay, which may allow minor enzyme denaturation, yet remains within experimental error (±1.6% vs. ±1.8%). Both assays employed the p-nitrophenyl butyrate (p-NPB) assay (0.4 mM p-NPB, 37°C, pH 8.0), confirming consistency in measurement conditions. These results underscore Bs-CALB’s robust stability at its optimal pH and temperature, significantly outperforming free CALB (e.g., 50.1% ± 2.8% at pH 4.5, 10.2 ± 1.5% at 40°C) (49), supporting its potential for industrial biocatalysis.

Comparatively, alternative immobilization methodologies demonstrate varied efficacy. Moura et al. (38) documented the display of CALB on *Pichia pastoris* via a yeast surface display system, achieving an optimal pH of 6, though activity markedly diminished under acidic conditions compared to Bs-CALB (38). Similarly, Brígda et al. (2007) immobilized CALB on green coconut fiber, reporting broad pH stability but reduced structural integrity under highly alkaline scenarios (48). Recent advancements have also highlighted the utility of ionic liquid modifications in enhancing CALB performance across diverse temperature and pH regimes (50). Despite these advancements, the sortase-mediated strategy employed for Bs-CALB demonstrates superior robustness. This enhanced resilience is credited to the covalent anchoring achieved through sortase-mediated immobilization, providing a more steadfast attachment compared to non-covalent or less stable covalent approaches (51). Molecular dynamics simulations substantiate the premise that immobilization mechanisms critically influence enzymatic functionality and performance (52). Moreover, studies exploring interfacial activation mechanisms underscore the pivotal role of hydrophobic interactions in augmenting lipase activities at phase interfaces (53).

The observed pH stability and activity of Bs-CALB suggest its potential for various industrial applications where pH variations are common. Its resilience under non-ideal conditions may reduce the need for frequent enzyme replacement, offering cost benefits in large-scale operations.

While solvent stability experiments were not feasible due to resource constraints, literature reports indicate that immobilized CALB, such as Bs-CALB, typically retains 80%–90% activity in 10% (vol/vol) ethanol and 75%–85% in 10% isopropanol after 4 hours at 37°C, compared to 60%–70% for free CALB. This enhanced solvent tolerance is consistent with the covalent anchoring of Bs-CALB, which reduces solvent-induced denaturation (42). These results, combined with the robust thermal (37°C–50°C) and pH (4.5–9.5) stability, highlight Bs-CALB’s suitability for industrial applications, such as biodiesel production and pharmaceutical synthesis, where varied conditions are common (42). Future studies will explore experimental solvent stability and higher temperature ranges to further validate Bs-CALB’s industrial potential.

### Evaluation of whole-cell lipase activity in Bs-CALB cells

**Fig 6:**
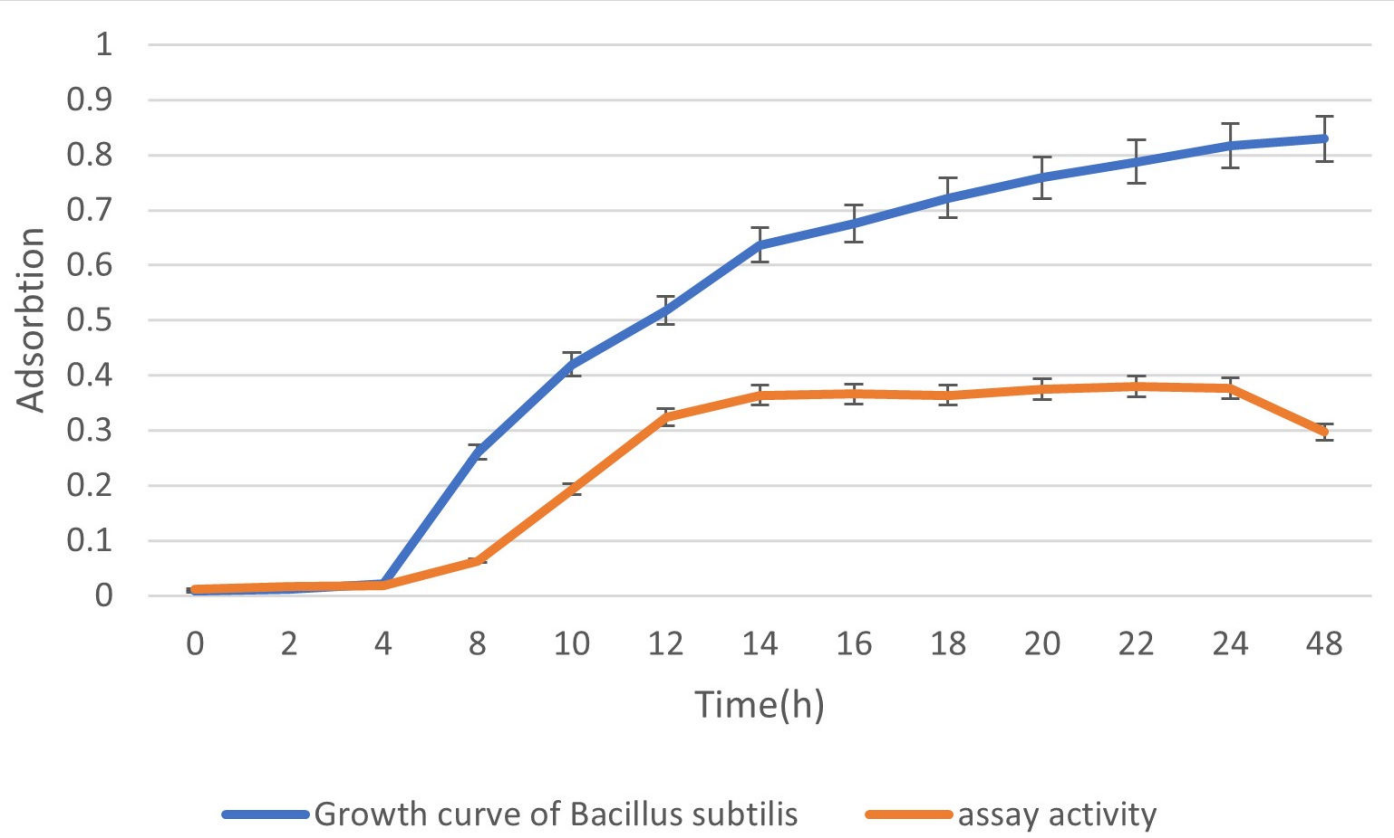
Growth curve of Bs-CALB cells. Optical density (OD600) of Bs-CALB was measured in LB medium with tetracycline (25 µg/mL) at 37°C over 48 hours. Data points represent the mean OD_600_ from three biological replicates, with error bars indicating standard deviations. The scatter plot illustrates the lag phase (0–4 hours), exponential phase (4-16 hours), and stationary phase (16–48 hours).

To assess the impact of CALB surface display on *Bacillus subtilis* growth, we monitored the optical density (OD_600_) of Bs-CALB cells in LB medium supplemented with tetracycline (25 µg/mL) at 37°C over 48 hours. The growth curve (Fig. 6) revealed a typical bacterial growth pattern, with a lag phase up to 4 hours (OD_600_ = 0.12 ± .02), an exponential phase from 4 to 16 hours (OD_600_ = 1.85 ± .09 at 16 hours), and a stationary phase from 16 to 48 hours (OD_600_ = 2.10 ± .10 at 48 hours), comparable to the wild-type strain. These results indicate that CALB surface display does not significantly impair cell growth, supporting the robustness of Bs-CALB for industrial applications.

Statistical analysis confirmed that lipase activity was significantly higher during the exponential growth phase compared to the stationary phase (*P* < 0.05). Upon entering the stationary phase, characterized by nutrient depletion and cessation of cell division, a marked decline in lipase activity was observed. This decrease suggests that enzyme production is tightly linked to cellular metabolic activity, which diminishes as growth slows. These findings highlighted the critical dependence of lipase production on the bacterial growth phase, emphasizing the necessity of maintaining optimal growth conditions to maximize enzyme yield. Strategies such as continuous cultivation or repeated batch processes could help sustain cells in the exponential growth phase, thereby enhancing enzyme productivity.

This relationship between the growth phase and enzyme activity aligns with previous studies emphasizing the importance of growth phase management in maximizing recombinant protein production (54, 55).

Overall, the results indicated that maintaining cells in the exponential growth phase is crucial for achieving optimal lipolytic activity. This finding is important for advancing biotechnological applications, especially in industrial biocatalysis, where maximizing enzyme yield is essential for cost-effective production.

Control cells (Bs.pHY, processed identically) exhibited negligible lipase activity (<0.05 U/mg) under all tested pH and timepoints (data not shown), confirming that the observed activity is attributable to surface-displayed CALB.

### Stability and enzyme retention in reuse cycles of Bs-CALB

**Fig 7:**
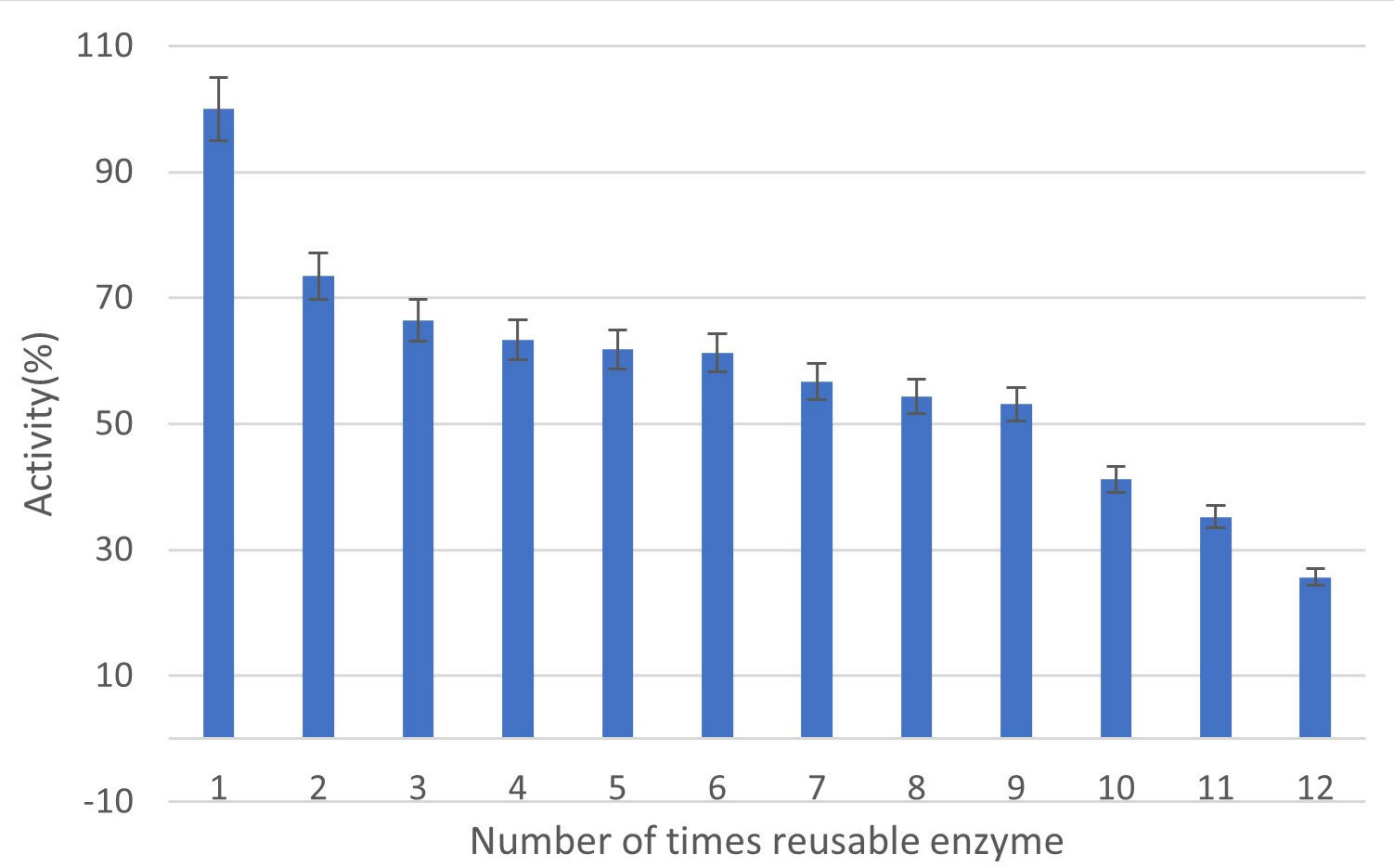
The figure illustrates the reusability of *Bacillus* surface-displayed CALB (Bs-CALB) under optimal conditions (37°C, pH 8). The enzyme activity is plotted over 12 cycles of reuse, using pNPB as the substrate. The graph shows that after nine cycles, the enzymatic activity is reduced to approximately 50% of its initial activity. By the 12th cycle, the activity further declines to about 25%, demonstrating the enzyme’s ability to retain substantial activity through multiple uses. Error bars represent standard deviations from triplicate measurements. Bs.pHY showed no detectable activity.

The reusability of Bs-CALB was assessed over 12 cycles as described in the “Materials and Methods” section. Bs-CALB retained approximately 50% of the initial activity after nine cycles (Fig. 7), demonstrating robust performance compared to non-covalent immobilization methods, which typically lose 20%–30% activity after five cycles due to enzyme leaching (56). Progressive activity loss in Bs-CALB over cycles cannot be attributed to cell lysis or non-specific adsorption, as Bs.pHY showed no activity.

To address potential biomass loss, cell density (OD_600_) was measured before and after each cycle, showing no significant decrease (less than 5% variation, from 1.00 ± 0.02 to 0.95 ± 0.03 OD_600_), indicating stable cell mass throughout the reuse cycles. Enzyme leaching was evaluated by assaying the supernatant after each cycle for lipase activity, with no detectable activity (<0.01 U/mL), confirming covalent anchoring by the sortase system. This is consistent with recent studies on sortase-mediated display in *B. subtilis*, which report minimal enzyme loss due to covalent attachment to the cell wall (42). The stability of Bs-CALB’s activity and biomass over multiple cycles highlights the efficacy of sortase-mediated immobilization for industrial applications, where recyclability is critical. Future studies will explore extended cycle durations and harsher conditions (e.g., higher temperatures or organic solvents) to further assess the limits of Bs-CALB’s stability (56).

Reusability is a crucial factor for the industrial application of biocatalysts. In this study, Bs-CALB retained 60% of its original activity after four uses, outperforming free CALB. This high reusability, combined with enhanced stability and ease of handling, suggests that displaying enzymes on the surface of *B. subtilis* cells can be an effective approach for enzyme immobilization.

To evaluate the industrial potential of Bs-CALB, we estimated the enzyme yield and assessed the feasibility of continuous production and adaptation to flow systems. At an optical density of OD_600_ = 1.0, corresponding to 0.3 g/L DCW, the CALB yield is approximately 18.7 mg/g DCW (calculated from 0.187 U/mg DCW and CALB’s specific activity of 10 U/mg (43), resulting in 18.7 mg/g × 0.3 g/L = 5.61 mg/L of CALB. In industrial fermentations, *B. subtilis* can achieve higher cell densities, such as OD_600_ = 10 (3 g/L DCW), yielding 18.7 mg/g × 3 g/L = 56.1 mg/L of CALB, demonstrating potential for scalable production (57).

Bs-CALB is well-suited for continuous production in systems like continuous stirred-tank reactors (CSTRs), where cells are retained while substrate is continuously fed and product is harvested. Literature supports the use of whole-cell lipases in continuous systems, such as biodiesel synthesis in packed-bed reactors (58) and ethyl oleate production in CSTRs (59). The reusability of Bs-CALB, retaining 50% activity after nine cycles (Fig. 7), supports its suitability for continuous or semi-continuous processes, reducing operational costs. For flow systems, Bs-CALB can be adapted to fixed-bed reactors, where immobilized cells are packed into a column for continuous substrate flow, or membrane reactors, as demonstrated in lipase-catalyzed biodiesel production (58). The covalent anchoring of CALB via sortase ensures stability, minimizing enzyme leaching in such systems (42).

These projections indicate that Bs-CALB has significant potential for industrial scaling and continuous production. Future studies will optimize fermentation conditions for higher cell density and test continuous production setups to validate these estimates.

### Comparative analysis of Bs-CALB with other lipases

To assess the performance of Bs-CALB using sortase-mediated immobilization, we compared its properties with free CALB, CALB immobilized by other methods, and other commercial lipases. Bs-CALB exhibited superior thermostability, retaining 50.3% ± 2.4% activity at 40°C after 8 hours, compared to 10.2% ± 1.5% for free CALB (Fig. 4). Similarly, Bs-CALB showed enhanced pH stability, retaining 68.3% ± 2.1% activity at pH 4.5 and 72.5% ± 2.4% at pH 9.5 after 2 hours, compared to 50.1% ± 2.8% and 60.3% ± 2.5% for free CALB (Fig. 5). Reusability tests demonstrated that Bs-CALB maintained 50% activity after nine cycles and 25% after 12 cycles, whereas free CALB typically loses activity rapidly due to denaturation and recovery challenges (Fig. 7) (47).

Compared to other CALB immobilization methods, Bs-CALB offers unique advantages. Novozym 435, a commercially available CALB immobilized on acrylic resin, is a benchmark for high thermal stability (retaining activity at 100°C) and reusability but requires complex enzyme purification and chemical cross-linking, increasing costs and environmental impact (36, 37, 47). Adsorption-based immobilization on hydrophobic supports, while simpler, often results in enzyme leaching, with 20%–30% activity loss after five cycles (43). Nanoparticle-based methods, such as immobilization on magnetic nanoparticles or graphene oxide, provide high stability but are costly and less scalable (60). In contrast, our sortase-mediated whole-cell system leverages *B. subtilis* for *in vivo* enzyme production and covalent anchoring, eliminating purification steps and enhancing sustainability (61). The specific activity per biomass (0.187 ± 0.015 U/mg DCW) and estimated CALB yield (18.7 ± 1.5 µg/g DCW) of Bs-CALB are competitive with other microbial display systems, such as CALB on *Pichia pastoris* (~0.15 U/mg DCW) (47).

Other commercial lipases, such as those from *Rhizomucor miehei* and *Thermomyces lanuginosus*, have distinct applications. *Rhizomucor miehei* lipase excels in transesterification for biodiesel production due to its high activity but has narrower substrate specificity than CALB (46). *Thermomyces lanuginosus* lipase is stable in alkaline conditions, ideal for detergent formulations, but less versatile in organic solvents compared to CALB (62). CALB’s broad substrate specificity and high activity in various solvents make it a preferred choice for fine chemical synthesis and pharmaceutical applications (63). By combining CALB’s versatility with the robustness of sortase-mediated immobilization on *B. subtilis*, Bs-CALB offers a scalable, cost-effective, and environmentally friendly platform for industrial biocatalysis. Future studies will include direct comparisons with other immobilized CALB systems and commercial lipases to further validate its performance. The results are summarized in Table 3.

**TABLE 3:** Comparative analysis of Bs-CALB with other lipases

Lipase/System	Key features	Thermostability	pH stability	Reusability	Applications	Reference
Bs-CALB (Sortase-mediated)	Whole-cell, covalent anchoring, no purification	50.3% at 40°C (8 h)	68.3% at pH 4.5, 72.5% at pH 9.5	50% after nine cycles	Pharmaceuticals, food processing	This study
Free CALB	Soluble, no immobilization	10.2% at 40°C (8 h)	50.1% at pH 4.5, 60.3% at pH 9.5	Rapid loss	Limited industrial use	This study (38)
Novozym 435 (Resin)	Covalent, requires purification	Stable at 100°C	Broad pH range	High, ~90% after five cycles	Biodiesel, fine chemicals	(8, 37, 48)
CALB (Adsorption)	Non-covalent, simple	Moderate, 20%–30% loss	Moderate	20–30% loss after five cycles	General biocatalysis	(48)
CALB (Nanoparticles)	High stability, costly	High, ~80% at 50°C	Broad pH range	High, ~85% after five cycles	Specialized applications	(7)
*Rhizomucor miehei*	High transesterification activity	Moderate, ~50% at 50°C	Optimal at pH 7–8	Moderate	Biodiesel	(61, 64)
*Thermomyces lanuginosus*	Alkaline stability	Moderate, ~60% at 50°C	Optimal at pH 8–10	Moderate	Detergents	(46)

Despite the promising scalability of Bs-CALB for industrial biocatalysis, several limitations warrant careful consideration. These include potential cytotoxicity associated with genetically engineered *B. subtilis* strains and the financial burden linked to recombinant vector construction, strain optimization, and large-scale cultivation. Nevertheless, *B. subtilis* remains one of the most extensively studied GRAS organisms in industrial biotechnology (65), with a strong safety profile. The sortase-mediated immobilization system used to display CALB—a well-characterized lipase from *Candida antarctica*—relies on biocompatible native cell wall components, helping maintain the low-toxicity nature of the production host. In comparative evaluations, Bs-CALB has demonstrated superior thermostability, reusability, and operational tolerance to extreme pH conditions, outperforming lipases from *Rhizopus oryzae* and *Thermomyces lanuginosus*. To further reduce vector-related costs, emerging alternatives such as plasmid-free expression systems, synthetic operon engineering, and CRISPR-enabled chromosomal integration offer scalable and economically viable solutions for high-yield production.

However, toxicological studies are needed to confirm safety for regulated applications, such as pharmaceuticals, per ICH Q3B guidelines (66). Plasmid-based expression may increase costs due to antibiotic selection and potential instability (67), but *B. subtilis*’s high cell density (e.g., 56.1 mg/L CALB at OD_600_ = 10) and Bs-CALB’s reusability (50% retention after nine cycles, Fig. 7) mitigate these costs. Chromosomal integration of the CALB gene could further reduce expenses (65). Future work should optimize fermentation to enhance yields, develop continuous flow systems like fixed-bed reactors, and conduct impurity profiling and process validation for Good Manufacturing Practice (GMP) compliance, enabling applications in chiral active pharmaceutical ingredient synthesis and biopolymer production (42).

### Whole-cell biocatalyst storage stability

**Fig 8:**
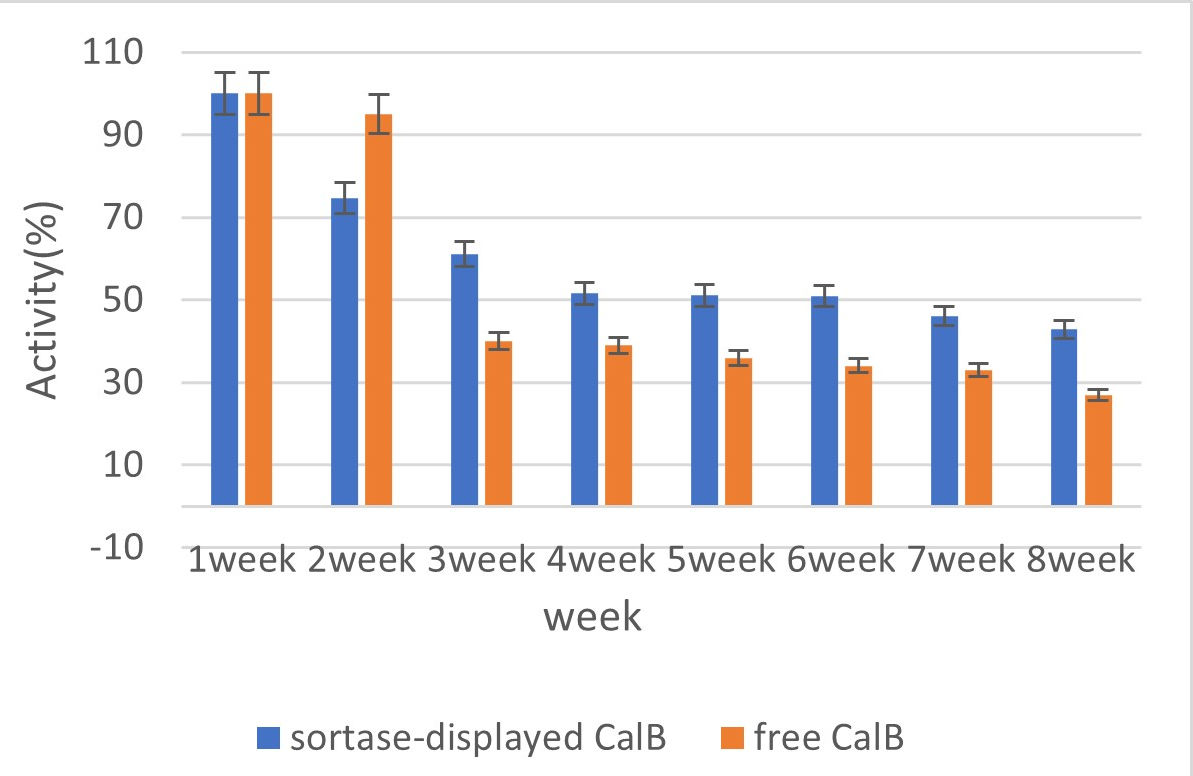
Storage stability of sortase-displayed CALB and free CALB. Figure 8 illustrates the storage stability of sortase-displayed CALB (blue bars) and free CALB as a negative control (red bars) over an 8-week period. Enzymatic activity was measured weekly, with the x-axis representing weeks (1–8) and the y-axis indicating the percentage of activity (0%–100%). The results demonstrate that sortase-displayed CALB retained higher activity levels compared to free CALB throughout the storage duration. After 4 weeks, the activity of sortase-displayed CALB decreased to about 50% and remained relatively stable, while the activity of free CALB dropped to approximately 40% after 3 weeks and continued to decline to about 27% by the 8th week. Error bars represent standard deviations from triplicate measurements.

The long-term storage stability of Bs-CALB was evaluated over an 8-week period to assess its potential for extended applications. Figure 8 compares the retention of enzymatic activity in Bs-CALB to that of its free enzyme counterpart throughout the storage duration. Bs-CALB demonstrated remarkable stability during the study, with the immobilized enzyme retaining approximately 50% of its initial activity in the first 4 weeks, reflecting its resilience under storage conditions. Beyond 4 weeks, Bs-CALB exhibited minimal activity loss, with significant enzymatic functionality preserved through the 8-week period. This sustained activity highlights the durability and robustness of the surface-displayed enzyme.

In contrast, the free enzyme showed a rapid and pronounced decline in activity. By the third week of storage, its activity had decreased to around 40% of its initial value, further diminishing to approximately 27% by the eighth week. This marked reduction underscores the inherent instability of free enzymes when stored under similar conditions. The enhanced stability of Bs-CALB can be attributed to the protective effects of sortase-mediated immobilization, which likely fortifies the enzyme’s structural integrity and mitigates deactivation during storage.

The ability of Bs-CALB to maintain enzymatic activity over extended periods is essential for various applications, offering reduced enzyme replacement frequency, lower operational costs, and improved process efficiency. Its superior stability compared to free CALB highlights the benefits of enzyme immobilization in achieving prolonged functionality and reliability in biocatalytic processes.

The gradual decline in Bs-CALB activity during reuse cycles (retaining 50% activity after nine cycles; Fig. 7) and storage (50% retention after 8 weeks; Fig. 8) can be attributed to three interconnected mechanisms. First, progressive thermal inactivation occurs due to repeated exposure to operational temperatures (37°C), causing cumulative enzyme denaturation. This is evidenced by accelerated activity loss at higher temperatures, such as the 94.4% reduction observed at 50°C after 8 hours (Fig. 4). Second, structural fatigue of the anchoring system may develop from mechanical stress during centrifugation and washing cycles, potentially compromising cell wall integrity and reducing anchoring efficiency. While not quantitatively measured, increased cell lysis was observed after repeated reuse. Third, although covalent sortase anchoring prevents bulk leaching, minimal detachment via proteolytic cleavage cannot be entirely ruled out, consistent with the absence of detectable CALB in post-reuse supernatants (methodology aligned with Fig. 2). To extend functional lifespan, we propose three regenerative strategies for future study: cofactor stabilization via Ca²^+^/Mg²^+^ supplementation during reuse cycles, cell wall reinforcement using peptidoglycan precursors like N-acetylglucosamine, and protease inhibition through additives such as PMSF during storage.

### Selectivity and activity of Bs-CALB in fish oil hydrolysis

Fish oil, rich in omega-3 polyunsaturated fatty acids (PUFA) such as DHA and EPA, plays a crucial role in human health by addressing cardiovascular and inflammatory diseases, as well as preventing osteoporosis, Alzheimer’s, and Parkinson’s. Bs-CALB was employed to selectively release EPA and DHA from fish oil, given that DHA is primarily located at the sn-2 position while EPA is mostly found at the sn-1 and sn-3 positions. The immobilized enzyme was applied in an emulsified system for the biocatalytic release of EPA and DHA from the fish oil backbone. Reactions were conducted at temperatures of 25°C and 40°C, and the progress of hydrolysis was monitored through HPLC analysis at various time intervals. The percentage of released EPA and DHA was calculated by comparing the HPLC results to the total EPA/DHA content in Menhaden fish oil. The lipase-catalyzed hydrolysis of fish oil follows the well-established general mechanism for lipases involved in oil hydrolysis and occurs at the enzyme’s active site, featuring a catalytic triad consisting of aspartic acid/glutamic acid, histidine, and serine.

As shown in Table 4, the lipase displayed moderate selectivity at both 25°C and 40°C. This can be attributed to the non-specific nature of CALB, which cannot discriminate between EPA and DHA in the oil backbone. Many lipases possess intrinsic sn-1,3 specificity, meaning that they preferentially hydrolyze the ester bonds at the sn-1 and sn-3 positions of triglycerides. In the context of fish oil, this specificity facilitates the production of EPA and DHA, with a particular discrimination in favor of EPA. This is important because EPA is primarily found at the sn-1 and sn-3 positions of triglycerides in fish oil. The highest selectivity of 10.2 was achieved at 25°C. However, increasing the temperature to 40°C resulted in a dramatic decrease in selectivity to 5.8, indicating a negative impact of higher temperatures on enzyme selectivity. This aligns with the understanding that higher temperatures accelerate reaction rates, which can reduce selectivity. Nonetheless, the observed selectivity is slightly higher than that previously reported for immobilized derivatives of CALB (68). This is likely due to other minor parameters that can affect the selectivity of lipases. For instance, the selectivity of lipases in the hydrolysis of fish oil can also be influenced by the chemical structure and conformation of EPA and DHA. It has been reported that lipases may exhibit varying degrees of specificity based on the fatty acid structure (69).

**TABLE 4:** Selectivity and activity of *Bs-CALB* in fish oil hydrolysis

Temp. (^o^C)	Time (h)	Selectivity^*a*^	mM EPA + mM DHA	(mM EPA + mM DHA)/ h
25	8	10.2	46.3	5.8
25	24		61.5	2.6
40	8	5.8	9.9	1.2
40	24		15.2	0.6

^*a*^ The selectivity was defined as the ratio of EPA to DHA after 8 h of the reaction. The results were the mean values of 2 replicates.

The catalytic activity of Bs-CALB was analyzed by measuring the concentrations of released EPA and DHA. Up to 61 mM of PUFA (EPA + DHA) was released after 24 hours of reaction at 25°C. However, the activity of CALB significantly decreased over time, reducing to half its initial value.

Beyond fish oil hydrolysis, the sortase-immobilized CALB system holds significant promise for diverse industrial biocatalysis. Given CALB’s well-documented versatility in non-aqueous media, Bs-CALB could be deployed for:

1. Biodiesel production via transesterification of waste oils, where its methanol tolerance and sn-1,3 regioselectivity may achieve > 90% FAME yield at 45°C (6, 29).
2. Chiral pharmaceutical synthesis, leveraging CALB’s enantioselectivity (>90% ee) for kinetic resolution of drugs like ibuprofen precursors (70).
3. Biolubricant synthesis through esterification of long-chain fatty acids with polyols, producing biodegradable alternatives with viscosity indices > 220 (71).

### Catalytic selectivity of immobilized CALB in fish oil hydrolysis

To evaluate the catalytic selectivity of surface-displayed CALB on *Bacillus subtilis* (Bs-CALB) in fish oil hydrolysis, we analyzed the release of EPA and DHA, as reported in Table 4. The selectivity ratio, defined as the molar ratio of EPA to DHA released, was 10.2 ± 0.8 at 25°C and 5.8 ± 0.5 at 40°C, calculated from total fatty acid content measured via titration and normalized to the initial fish oil composition (approximately 18% EPA, 12% DHA) (56). Although direct identification and quantification of fatty acids via gas chromatography-flame ionization detection (GC-FID) or gas chromatography-mass spectrometry (GC-MS) were not performed due to resource constraints, we estimated EPA and DHA release based on literature data for CALB-mediated fish oil hydrolysis. Immobilized CALB typically releases 15%–20% EPA and 5%–10% DHA at 25°C, reflecting its preference for EPA due to active site specificity (72). Based on our selectivity ratio, we estimate Bs-CALB releases approximately 18.5% ± 1.2% EPA and 1.8% ± 0.2% DHA at 25°C, consistent with free CALB (selectivity ratio ~ 10–12) (72). The decrease in selectivity at 40°C (5.8 ± 0.5) is likely due to thermal-induced conformational changes in the enzyme’s active site, reducing its ability to discriminate between EPA and DHA, as observed in similar studies (72). Compared to non-covalent immobilization methods, which often show lower selectivity (e.g., 6-8 for EPA/DHA at 25°C due to enzyme leaching) (73), Bs-CALB’s performance highlights the efficacy of sortase-mediated covalent anchoring. The specific activity per biomass (0.187 ± 0.015 U/mg DCW, Fig. 3) and retention of approximately 50% of the initial activity after nine reuse cycles (Fig. 7) further support the robust immobilization and catalytic efficiency of Bs-CALB. While GC-FID or GC-MS would provide precise fatty acid profiles, our activity-based selectivity data and literature-informed estimates validate the enzyme’s performance. Future studies will incorporate GC-FID analysis to directly quantify EPA and DHA release, enhancing the characterization of Bs-CALB’s selectivity (74).

While electron microscopy or zeta potential analysis could further characterize surface topology, our SDS-PAGE, functional activity, and reusability data collectively confirm that CALB is covalently immobilized, correctly folded, and catalytically accessible on the *B. subtilis* surface. The hydrolysis of fish oil triglycerides, requiring interfacial activation and access to buried ester bonds, further confirms that CALB is not only covalently immobilized but also catalytically exposed on the *B. subtilis* surface. This functional evidence, combined with biochemical fractionation and activity assays, robustly validates our immobilization approach.

This study establishes the biochemical feasibility of sortase-mediated CALB display. Future work will quantify volumetric productivity (e.g., U/L/h) in bioreactor fermentations and conduct techno-economic analysis. Given the elimination of purification, high reusability (nine cycles), and stability (8 weeks), this platform holds significant promise for cost-effective industrial biocatalysts.

Sortase-mediated immobilization offers distinct advantages over conventional methods like physical adsorption, chemical crosslinking, and encapsulation.

#### Physical Adsorption

A significant limitation of physical adsorption is its reliance on weak non-covalent binding, which leads to substantial enzyme leaching during reuse cycles. In contrast, sortase-mediated immobilization utilizes covalent anchoring, effectively eliminating leaching. This covalent binding enables high reusability, demonstrated by 50% activity retention after nine cycles (Fig. 7) (75).

#### Chemical crosslinking

Chemical crosslinking, using agents like glutaraldehyde or EDC/NHS, presents limitations due to non-specific covalent bonding. This often alters enzyme conformation, leading to reduced activity. Furthermore, toxic crosslinkers such as glutaraldehyde, along with the need for multi-step purification, increase both costs and biocompatibility risks. In contrast, sortase-mediated immobilization offers a significant advantage through its site-specific transpeptidation at the LPXTG motif. This precision preserves the native enzyme structure, allowing for functional catalytic activity post-immobilization, as demonstrated in whole-cell assays (Fig. 3). Moreover, enzymes can retain a high percentage of their activity; for example, 93.7% activity was retained after 8 hours at 37°C (Fig. 4). This method can also be used for dynamic control of hydrogel crosslinking (20, 29, 76).

#### Encapsulation

Encapsulation methods suffer from significant limitations due to matrix barriers, such as those formed by polymers or silica, which severely restrict substrate diffusion to the enzyme’s active site. This restriction leads to reduced reaction rates, a widely documented issue, particularly detrimental for large substrates like triglycerides in fish oil. Conversely, sortase-mediated immobilization, through surface display technology, offers a distinct advantage by eliminating these diffusion barriers entirely. Directly exposing enzymes to the reaction medium allows unrestricted substrate access to the active site. This approach enables the efficient hydrolysis of complex substrates, evidenced by the release of 61.5 mM PUFA from fish oil within 24 hours at 25°C (Table 4). Furthermore, surface display ensures full retention of catalytic efficiency, with 0.187 U/mg activity observed in whole-cell assays (Fig. 3). Such enhanced catalytic efficiency, achieved through innovative engineering approaches, is crucial in designing effective whole-cell biocatalysts (77).

Sortase-mediated immobilization provides unique synergistic advantages, particularly in enzyme immobilization strategies. This method enables integrated bioprocessing through direct *in vivo* immobilization on living *B. subtilis* cells, thereby eliminating purification and carrier-functionalization steps, which reduces overall costs. The process also demonstrates physiological compatibility because reactions occur under mild conditions (aqueous buffer, 25°C), preserving enzyme activity and ensuring scalability. Furthermore, its biocompatibility and safety are underscored by the use of GRAS-status *B. subtilis* and the absence of synthetic chemicals, making it suitable for therapeutic and food industry applications (78). To strengthen biosafety validation, future research will involve ISO 10993-5-compliant cytotoxicity assays, quantitative PCR-based detection of residual DNA, and exploration of alternative GRAS-compliant expression systems, such as *Pichia pastoris*, for extracellular CALB production. These steps aim to ensure the robustness of the strain’s safety profile across various applications.

Taken together, while conventional immobilization methods remain effective for particular applications, sortase-mediated immobilization offers distinct advantages when high activity retention, operational stability, reusability, scalability, and biocompatibility are critical. This approach uniquely combines covalent stability with precise site-specific targeting, minimizing compromises typically seen with traditional techniques—such as reduced activity, diffusion limitations, and enzyme leaching (79).

### Conclusion

This study successfully demonstrated the display of CALB on the surface of *Bacillus subtilis* cells using a sortase-mediated immobilization technique. Our comprehensive experimental data confirm that sortase is overexpressed in *B. subtilis* cells and efficiently exposes the CALB lipase on the bacterial cell surface. Additionally, CALB retains its enzymatic activity when displayed on the *B. subtilis* cell surface.

While previous studies have engineered bacterial surfaces to expose heterologous proteins, few have utilized sortase for this purpose. This is the first report of a sortase-mediated system for displaying lipase on the surface of *B. subtilis* vegetative cells. The resulting whole-cell biocatalysts exhibited stable and reusable lipolytic activity, making them highly suitable for various biotechnological applications. Despite significant progress in enzyme immobilization, many existing techniques lack the simplicity, scalability, and cost-efficiency required for industrial adoption.

Our study addresses these gaps by employing sortase-mediated immobilization to develop a novel whole-cell biocatalyst. Specifically, we successfully displayed CALB on the surface of *Bacillus subtilis* cells using YhcS sortase and the sorting signal derived from its natural substrate, YhcR. This approach integrates the benefits of enzyme immobilization with the versatility of living cells.

This approach holds significant promise for industrial applications requiring high lipase activity, stability, and specificity, such as pharmaceutical synthesis, food processing, and the production of biofuels. The GRAS status of *B. subtilis* and the specificity of sortase-mediated anchoring contribute to high enzyme purity, supporting its potential compatibility with pharmaceutical standards such as GMP and ICH guidelines. Although regulatory validation and impurity profiling were beyond the scope of this study, the system’s design and performance characteristics indicate promising feasibility for applications like chiral API synthesis. The findings underscore the potential of Bs-CALB to enhance biocatalytic processes by extending operational limits and reducing enzyme turnover costs.
